# Robot-assisted, minimally invasive spinal tumor resection and posterior spinal fusion

**DOI:** 10.3171/2026.4.FOCVID25225

**Published:** 2026-07-01

**Authors:** Alexa Semonche, Rahul Sastry, Cecilia Dalle Ore, Robert C. Osorio, Conor Grady, Alekos Theologis, Aaron J. Clark

**Affiliations:** 1 Department of Neurological Surgery, University of California, San Francisco; and; 2 Department of Orthopaedic Surgery, University of California, San Francisco, California

**Keywords:** robotic spine surgery, minimally invasive spine surgery, spinal metastases

## Abstract

Integrating robotics with neuronavigation and tubular dilation enables minimally invasive posterior stabilization and tumor resection. A 40-year-old woman presented with diffuse spinal metastases with spinal cord compression. This surgical video demonstrates the authors’ entire minimally invasive workflow for robot-assisted percutaneous pedicle screw placement, arthrodesis, and navigated tubular resection. The authors review their institutional experience with 14 cases using this workflow. The advantages of this approach include the high accuracy of pedicle screw placement, minimal blood loss, and expedited time to postoperative chemoradiation therapy compared to traditional open, nonrobotic approaches.

The video can be found here: https://stream.cadmore.media/r10.3171/2026.4.FOCVID25225

## Transcript

This video demonstrates our workflow for robot-assisted, minimally invasive spinal tumor resection and posterior spinal fusion.

### 0:30 Clinical Presentation.

The patient is a 40-year-old female with a past medical history of beta-thalassemia and breast cancer who presented with 1 month of back pain and 2 weeks of progressive bilateral lower extremity weakness. She was unable to ambulate due to gait imbalance, but denied any bowel or bladder dysfunction. On exam, the patient had 4/5 strength in the proximal bilateral lower extremities and 4+/5 strength distally. There were no pathological reflexes.

### 0:58 Preoperative Imaging.

MRI imaging showed diffuse metastases throughout the spine and Bilsky grade 2 epidural compression of the spinal cord at T6 and T9. CT imaging of these levels demonstrates multiple pathologic fractures.

### 1:10 Surgical Plan.

Our surgical plan accounted for the patient’s medical frailty and need for urgent chemoradiation to her diffuse metastatic disease burden. To achieve this, we first asked our vascular neurosurgeon to perform a diagnostic angiogram and embolize arterial feeders to metastases where able. We also focused on medical optimization by correcting metabolic derangements and preparing typed and crossed blood to be available during surgery. The surgical plan was for a robot-assisted, minimally invasive T4–11 posterior spinal fusion with T6 and T9 laminectomies and partial corpectomies using the Mazor surgical robot, Stealth neuronavigation, and METRx tubular dilator set.

### 1:55 Planning Screw and Arthrodesis Trajectories, Patient-Specific Rods.

The Mazor planning software was used to plan trajectories for pedicle screws and facet decortication for arthrodesis. This planning step allows us to ensure the pedicle screws are aligned to facilitate percutaneous rod placement. This also enables us to order custom Medicrea rods specific to the patient and our target alignment.

### 2:18 Patient Positioning.

The patient was positioned prone with the head fixed in a Mayfield head holder. The Mazor robot was mounted to the operating table. The thoracic spine region was prepped and draped in standard fashion.

### 2:32 Robot Preparation.

Next, the robot is draped and the arm is brought into the surgical field. The instrument cannula and Stealth reference array are attached to the robot. A percutaneous pin is placed in the posterior superior iliac spine and attached to the robot arm.

### 2:56 Robot and Neuronavigation Registration.

The Stealth neuronavigation workstation is placed at the foot of the bed. Navigated instruments are registered to the reference array on the robot. The robot is registered to the patient through a series of fluoroscopic images and merging these with a preoperative thin-cut CT scan. The accuracy of the navigation is checked against known anatomical landmarks.

### 3:17 Posterior Spinal Fusion.

First, the robot arm is sent to planned trajectories for arthrodesis. Then, 1.5-cm skin incisions were made over the entry points, and the burr drill attachment was used to decorticate the right-sided facet joints from T4 to T11. The sites were then packed with allograft. The screenshot here illustrates the plan for one trajectory for facet decortication. We plan three total sites like this for each facet joint.

### 3:55 Pedicle Screw and K-Wire Placement.

The robot arm is then sent to the pedicle screw entry points. On the left side, pedicle screws are placed through 1.5-cm skin incisions except for T6 and T9. On the right side, screw trajectories were navigated and drilled, but only K-wires were inserted to mark these trajectories. We leave K-wires on this side to leave room for tubular dilation for decompression. Note that screw placement and facet decortication can be achieved through the same incision side. The screenshot here demonstrates the use of neuronavigation to place a pedicle screw along the preplanned trajectory.

### 4:21 Navigated Tubular Dilation.

Here we see that left-sided screws and right-sided K-wires are in position. Next, we used navigation to place another K-wire through the right-sided incision closest to the T6 pedicle and placed serial dilators over the wire, followed by a 22-mm METRx tube. The navigation wand was used to confirm the position of the tube. The T6 laminectomy and transpedicular partial corpectomy will take place through this tube. An operating microscope is brought into the field.

### 4:51 Tubular Laminectomy.

The decompression at T6 began with removal of soft tissue overlying the lamina. A high-speed burr was used to expose the ligamentum flavum. This "over-the-top" decompression technique allowed us to remove the entire lamina from a left-sided approach. The ligamentum flavum was then resected using a blunt hook and Kerrison punches.

### 5:25 Transpedicular Decompression, Partial Corpectomy.

Next, the high-speed burr was used to drill down the right T6 pedicle. After thinning the walls of the pedicle, a curette was used to dissect the walls free from the thecal sac. Then, the pedicle was resected. The partial corpectomy was then completed using the drill, downpushing curettes, and pituitary rongeurs. Intermittently, we used neuronavigation to check the extent of the corpectomy. A nerve hook was passed underneath the thecal sac and nerve roots to confirm decompression of the neural elements was complete.

### 6:19 Hemostasis and Tubular Dilator Removal.

Then, hemostasis was achieved with fibrillar, bipolar cautery, and gelatin hemostatic matrix. As the METRx tube is removed, you can see the paraspinal muscles falling back into view.

### 6:49 Rodding.

Tubular decompression was repeated at T9. Then, the right-sided screws were placed over their K-wires and K-wires were removed. Although not shown here, vertebroplasties at T4 and T11 were also performed. Then, 5.5-mm titanium rods were then passed percutaneously through the screw heads and secured with locking caps. Under fluoroscopy, a cantilever technique was used to reduce the T6 and T9 pathological fractures and reduce the associated kyphosis.

### 7:20 Final Intraoperative Imaging.

Final fluoroscopy shots confirmed appropriate position of all instrumentation. Set screws were final tightened. Note that radiolucent carbon fiber screws were placed at levels adjacent to T6 and T9 to reduce imaging artifact for subsequent radiation treatment planning. A subfascial drain was placed and the skin incisions were closed in layers.

### 7:41 Clinical Outcome.

The patient’s neurological exam remained stable postoperatively. Our minimally invasive approach enabled expedited radiation therapy starting on postoperative day 6 and anastrozole on postoperative day 9. In comparison, at our institution, patients undergoing open spine procedures are not able to start chemoradiation until postoperative day 14. The patient did have postoperative anemia requiring blood transfusions in the setting of her known beta-thalassemia. The estimated blood loss for our procedure was 1 L, and an open surgical approach would likely have more. The patient was discharged to a skilled nursing facility on postoperative day 30.

### 8:19 Institutional Experience.

To date, we have performed 14 procedures using this workflow for patients with spinal tumors. This table summarizes our institutional experience and is available to review in Supplementary Table 1. Briefly, most patients underwent preoperative angiogram and embolization, and half of these patients were able to start postoperative chemoradiation sooner than postoperative day 14. The estimated blood loss ranged from 50 mL to 1 L.

### 8:44 Final Thoughts.

This video demonstrates a minimally invasive, robot-assisted approach to spinal tumor surgery, which has several advantages. Robotic assistance optimizes pedicle screw placement accuracy compared to freehand or traditional navigation techniques. We are still able to achieve arthrodesis percutaneously by planning separate robot trajectories for facet decortication. When combined with neuronavigation and MIS tubular techniques, our workflow provides a minimally invasive surgical corridor to epidural and bony metastatic disease for decompression. Similar to other published findings, we find that this workflow minimizes blood loss, radiation exposure to patient and staff, and expedites the time to postoperative chemoradiation.^1–8^ Finally, we find that incorporating preoperative angiogram and embolization and intraoperative cement augmentation are useful adjuncts.^9,10^ While the individual components of this procedure have been reported previously, this video synthesizes an entirely minimally invasive workflow for this pathology.

## Disclosures

A. Clark reported personal fees from Alphatec, Carlsmed, Icotec, and Medtronic outside the submitted work.

## Author Contributions

Primary surgeon: Theologis, Clark. Assistant surgeon: Sastry, Dalle Ore. Editing and drafting the video and abstract: Semonche, Sastry, Osorio, Theologis, Clark. Critically revising the work: Semonche, Sastry, Osorio, Grady, Theologis, Clark. Reviewed submitted version of the work: all authors. Approved the final version of the work on behalf of all authors: Semonche. Supervision: Theologis, Clark.

## Supplemental Information

Supplementary Table 1

### Online-Only Content

Supplemental material is available online.

*Supplementary Table 1.* https://thejns.org/doi/suppl/10.3171/2026.4.FOCVID25225.

### Patient Informed Consent

The necessary patient informed consent was obtained in this study.
