# Supplementary material for: Robot-assisted, minimally invasive spinal tumor resection and posterior spinal fusion
Source: Neurosurg Focus Video. 2026 Jul 1;15(1):V12. doi: 10.3171/2026.4.FOCVID25225 (PMC13418642; doi:10.3171/2026.4.FOCVID25225)
Supplement: Supplementary Table 1 [file SupplementaryTable1_FOCVID25-225.pdf]

NEUROSURGICAL FOCUS VIDEO

ONLINE ONLY

Supplemental material

Robot-assisted, minimally invasive spinal tumor resection and posterior spinal fusion

Semonche et al.

https://thejns.org/doi/abs/10.3171/2026.4.FOCVID25225

DISCLAIMER The *Journal of Neurosurgery* acknowledges that the following section is published verbatim as submitted by the authors and did not go through either the *Journal's* peer-review or editing process.

© 2026 The authors, CC BY 4.0 (http://creativecommons.org/licenses/by/4.0/)

Supplementary Table 1. Single institution experience of 14 robot-assisted MIS posterior spinal posterior arthrodesis and decompression for tumor.

Case	Age/Sex	Surgery	Pathology	Preoperative angiogram, embolization	Preoperative ASA grade	Estimated blood loss (mL)	Postoperative chemotherapy or radiation while inpatient	Complication	Disposition
1	68M	T2-6 posterior arthrodesis, T3-5 laminectomies, T3-5 tumor resection	Metastatic carcinoma consistent with prostate primary	Y	3	300		-	ARU
2	59F	T8-12 posterior arthrodesis, right T10-11 hemi-laminectomies, partial T10 corpectomy, T9-11 tumor resection	Metastatic carcinoma consistent with thyroid origin	Y	2	500	POD8, XRT	-	Home
3	92F	T9-L1 posterior arthrodesis, T11 laminectomy, T9-10 and L1 kyphoplasty, T11 tumor resection	Lambda-restricted plasma cell neoplasm	Y	2	50	POD6, XRT	-	SNF
4	75M	L1-5 posterior arthrodesis, L1-5 vertebroplasties, L2-4 laminectomies	Alk-negative anaplastic large cell lymphoma	N	3	100	POD5, chemotherapy	-	Home
5	58F	T11-L3 posterior arthrodesis, partial L1 corpectomy, T12 lamiectomy, T11 and L3 vertebroplasty, T12-L2 tumor resection	Metastatic adenocarcinoma, consistent with breast origin	Y	3	300		-	Home
6	60M	T9-L1 posterior arthrodesis, T10-12 laminectomies, L1 vertebroplasty, T10-12 tumor resection	Metastatic poorly differentiated carcinoma	Y	3	100	POD24, chemotherapy	Acute respiratory failure due to pleural effusion, treated with re-intubation and thoracentesis	SNF

7	39F	T12-L4 posterior arthrodesis, L1-3 laminectomies, partial L2 corpectomy, resection of tumor L1-3	Metastatic squamous cell carcinoma	N	3	200	POD11, XRT	-	Home
8	80F	T8-10 posterior arthrodesis, T9 tumor resection, T8-10 laminectomies, T8 and T10 vertebroplasties	Metastatic adenocarcinoma, consistent with origin from pulmonary primary	Angiogram performed, no embolization	2	50		-	Home
9	61F	L2-pelvis posterior arthrodesis, L2 vertebroplasty	N/A, history of metastatic cervical cancer	N	3	200	POD7, XRT	-	ARU
10	44M	T10-L2 posterior arthrodesis, partial T12 corpectomy, T11-L1 laminectomies, tumor resection T11-L1	Metastatic colon adenocarcinoma	Y	3	100	POD10, chemotherapy	Shock liver, acute kidney injury due to hypotension, managed medically	ARU
11	40F	T4-11 posterior arthrodesis, T5-10 laminectomies, partial T9 corpectomy, tumor resection T5-10	Metastatic carcinoma consistent with ductal carcinoma of breast	Y	3	1000	POD6, XRT<br><br>POD9, chemotherapy	Hypoxemia due to pleural effusion, treated with diuresis, durotomy	SNF
12	28F	T4-6 posterior arthrodesis, T4-6 laminectomies, R T5 transpedicular tumor resection	Meningioma, CNS WHO grade 1	N	2	100		Compressive seroma causing paraplegia, return to OR for decompression and duraplasty	ARU
13	74F	T9-L1 posterior arthrodesis, T10-12 laminectomies, T10-L2 tumor resection	N/A, history of metastatic breast cancer	N	3	100		-	Home
14	69M	T8-12 posterior arthrodesis, T9-11 laminectomies, partial T10 corpectomy, T0-11 tumor resection	Metastatic poorly differentiated carcinoma	Angiogram performed, no embolization	3	500	POD6, XRT	-	Home
